# Supplementary material for: Juvenile honest food solicitation and parental investment as a life history strategy: A kin demographic selection model
Source: PLoS One. 2018 Mar 1;13(3):e0193420. doi: 10.1371/journal.pone.0193420 (PMC5832247; doi:10.1371/journal.pone.0193420)
Supplement: S1 Appendix — (PDF) [file pone.0193420.s001.pdf]

Supplementary Information

Juvenile honest food solicitation and parental investment as a life history strategy: a kin demographic selection model

József Garay[1,2*], Villő Csiszár[3], Tamás F. Móri[3], András Szilágyi[1,2], Zoltán Varga[4], Szabolcs Számadó[5,2]

[1] MTA-ELTE Research Group in Theoretical Biology and Evolutionary Ecology, Department of Plant Systematics, Ecology and Theoretical Biology, ELTE Eötvös Loránd University, Budapest, Hungary

[2] Evolutionary Systems Research Group, MTA Centre for Ecological Research, , Tihany, Hungary.

[3] Department of Probability Theory and Statistics, Eötvös Loránd University, Budapest, Hungary

[4] Department of Mathematics, Szent István University, Gödöllő, Hungary

[5] RECENS „Lendület" Research Group, MTA Centre for Social Science, Budapest, Hungary

* e-mail: garayj@caesar.elte.hu

Appendix 1. Details of the Markov chain model

If $0 < \alpha < 1$, then we model the feeding process with a Markov chain X_t, $t = 1, 2, ..., T$, where X_t denotes the state of the process just after the parent has given away the tth unit of food (i.e., the state of the process at time t). We will have a total of $d = 3n - 2$ different states, each state will be a pair (i, k), where i denotes the state of the focal nestling: it can be any of the three possibilities H (hungry), D (digesting previously received food) or R (it just received food). The second coordinate k records the number of digesting nestlings (either digesting food received earlier, or food received at this moment) among the remaining $n - 1$ ones. There are $n - 1$ states (H, k), $k = 1, ..., n - 1$, also $n - 1$ states (D, k), $k = 1, ..., n - 1$, and n states (R, k), $k = 0, 1, ..., n - 1$. We denote the state space by S. From the previous description of the process, it is immediate to write down the transition matrix P of the Markov chain X_t. The elements of P are the transition probabilities $P(u, v) = \mathrm{P}(X_{t+1} = v \mid X_t = u)$, where u, v are any two states. Let us denote the binomial probabilities by $p_\alpha(k, j) = \binom{k}{j}(1-\alpha)^j \alpha^{k-j}$ (this is the probability that out of k digesting nestlings, exactly j are still digesting at the next time step) and let $\beta_j = \frac{1}{n-j}$. Then the $(H, k)^{\text{th}}$ row of the transition matrix is:

$$P((H,k),(H,j)) = p_\alpha(k, j-1)(1-\beta_{j-1}), \quad 1 \le j \le \min(k+1, n-1)$$

$$P((H,k),(R,j)) = p_\alpha(k, j)\beta_j, \quad 0 \le j \le k,$$

and all other elements are zero. The $(R, k)^{\text{th}}$ row of the transition matrix for $k < n - 1$ is:

$$P((R,k),(H,j)) = \alpha p_\alpha(k, j-1)(1-\beta_{j-1}), \quad 1 \le j \le \min(k+1, n-1)$$

$$P((R,k),(R,j)) = \alpha p_\alpha(k, j)\beta_j, \quad 0 \le j \le k$$

$$P((R,k),(D,j)) = (1-\alpha)p_\alpha(k, j-1), \quad 1 \le j \le \min(k+1, n-1),$$

and all other elements are zero. The $(R, k)^{\text{th}}$ row of the transition matrix for $k = n - 1$ is of the same form, except that

$$P((R,n-1),(R,n-1)) = \alpha p_\alpha(n-1,n-1)\beta_{n-1} + p_\alpha(n,n)\beta_0,$$

and

$$P((R,n-1),(D,n-1)) = (1-\alpha)p_\alpha(n-1,n-2) + p_\alpha(n,n)(1-\beta_0),$$

where the additional terms cover the cases when none of the nestlings are hungry. Finally, the $(D, k)^{\text{th}}$ row of the transition matrix is exactly the same as the $(R, k)^{\text{th}}$ row.

It is clear that our Markov chain is irreducible, and thus it is positive recurrent admitting a unique stationary distribution $\pi = (\pi_s : s \in S)$. Let $R^* = \{(R, k): k = 0, 1, ..., n-1\}$ be the subset of the states in which the focal nestling receives the food. Then the focal nestling's accumulated amount of food Y_T is just the number of visits of the chain in R^* up to time T. By introducing the reward function on the states

$$r(s) = \begin{cases} 1, & s \in R^* \\ 0 & s \notin R^* \end{cases}$$

we can write $Y_T = \sum_{t=1}^{T} r(X_t)$. It is a standard result that the central limit theorem holds for Y_T. To formulate the precise statement, let Π be the $d \times d$ matrix whose $(u, v)^{\text{th}}$ entry is π_v, and Π_{dg} the $d \times d$ diagonal matrix whose $(u, u)^{\text{th}}$ entry is π_u, and all other entries are 0. The $d \times d$ identity matrix is denoted by I. Finally, let $Z = (I - P + \Pi)^{-1}$ be the so-called fundamental matrix of P.

Theorem 1. (Central limit theorem for Markov chains, e.g. [1], p. 84) Let X_t be an irreducible Markov chain with finite state space S, and r any reward function on the states. Let π be the unique stationary distribution of the chain, and define

$$\mu = \sum_{s \in S} r(s)\pi_s$$

the mean of the reward function under stationarity, and

$$\sigma^2 = r\Pi_{dg}(2Z - I - \Pi)r^T,$$

where $r = (r(s) : s \in S)$ is the row vector of the rewards. Then for any initial distribution of X_1,

$$\frac{\sum_{t=1}^{K} r(X_t) - K\mu}{\sqrt{K}\sigma} \to N(0,1), \quad K \to \infty$$

in distribution, where $N(0, 1)$ stands for the standard normal distribution.

Let A be the $d \times d$ matrix with all its entries equal to 1, and $\mathbf{1} = (1, ..., 1)$. Since $\pi = \mathbf{1}(I - P + A)^{-1}$, both μ and σ are straightforward to calculate from P by using matrix arithmetics.

In our case, as noted earlier,

$$\mu = \sum_{s \in S} r(s)\pi_s = \sum_{s \in R^*} \pi_s = \frac{1}{n},$$

since in the stationary scenario, each nestling acquires the food with the same probability.

References

1. Kemeny JG, Snell JL. Finite markov chains: van Nostrand Princeton, NJ; 1960.
